# Bi-specific autoantigen-T cell engagers as targeted immunotherapy for autoreactive B cell depletion in autoimmune diseases

**DOI:** 10.3389/fimmu.2024.1335998

**Published:** 2024-02-26

**Authors:** Luca Perico, Federica Casiraghi, Fabiane Sônego, Marta Todeschini, Daniela Corna, Domenico Cerullo, Anna Pezzotta, Patricia Isnard-Petit, Silvia Faravelli, Federico Forneris, Kader Thiam, Ariela Benigni, Giuseppe Remuzzi

**Affiliations:** ^1^ Department of Molecular Medicine, Istituto di Ricerche Farmacologiche Mario Negri IRCCS, Bergamo, Italy; ^2^ Preclinical Models & Services, genOway, Lyon, France; ^3^ The Armenise-Harvard Laboratory of Structural Biology, Department of Biology and Biotechnology, University of Pavia, Pavia, Italy

**Keywords:** autoimmune diseases, membranous nephropathy, anti-PLA2R antibodies, autoreactive B cell, targeted immunotherapies, bi-specific autoantigen-T cell engagers

## Abstract

**Introduction:**

In autoimmune diseases, autoreactive B cells comprise only the 0.1-0.5% of total circulating B cells. However, current first-line treatments rely on non-specific and general suppression of the immune system, exposing patients to severe side effects. For this reason, identification of targeted therapies for autoimmune diseases is an unmet clinical need.

**Methods:**

Here, we designed a novel class of immunotherapeutic molecules, Bi-specific AutoAntigen-T cell Engagers (BiAATEs), as a potential approach for targeting the small subset of autoreactive B cells. To test this approach, we focused on a prototype autoimmune disease of the kidney, membranous nephropathy (MN), in which phospholipase A_2_ receptor (PLA_2_R) serves as primary nephritogenic antigen. Specifically, we developed a BiAATE consisting of the immunodominant Cysteine-Rich (CysR) domain of PLA_2_R and the single-chain variable fragment (scFv) of an antibody against the T cell antigen CD3, connected by a small flexible linker.

**Results:**

BiAATE creates an immunological synapse between autoreactive B cells bearing an CysR-specific surface Ig^+^ and T cells. *Ex vivo*, the BiAATE successfully induced T cell-dependent depletion of PLA_2_R-specific B cells isolated form MN patients, sparing normal B cells. Systemic administration of BiAATE to mice transgenic for human CD3 reduced anti-PLA_2_R antibody levels following active immunization with PLA_2_R.

**Discussion:**

Should this approach be confirmed for other autoimmune diseases, BiAATEs could represent a promising off-the-shelf therapy for precision medicine in virtually all antibody-mediated autoimmune diseases for which the pathogenic autoantigen is known, leading to a paradigm shift in the treatment of these diseases.

## 1 Introduction

Autoimmune diseases are heterogeneous pathogenic conditions in which the immune system produces autoantibodies that attack healthy tissues. Given their chronic nature, autoimmune diseases can be life-threatening and are one of the leading causes of death in females of all age groups (1).

Current first-line treatments mainly rely on non-specific and general immunosuppression, such as corticosteroids and alkylating agents (2). As a major shortcoming, these therapies also suppress immune cells that are functioning normally, leaving patients exposed to infections and cancer.

In the field of oncology, several biologics have been developed to induce deep depletion of B cells in patients with B cell malignancies, such as the monoclonal antibody rituximab, which targets the specific antigen CD20 on B cells (3). In this context, our group was the first to report the beneficial effect of rituximab in the treatment of membranous nephropathy (MN), an autoimmune disease of the kidney (4, 5). MN is the most common leading causes of nephrotic syndrome in adults and can lead to end-stage renal disease, requiring renal replacement therapies (6). In this context, we documented that the long-term positive outcome after treatment with rituximab was associated with reduction in circulating IgG_4_ antibody against phospholipase A_2_ receptor (PLA_2_R) (7, 8), the major autoantigen in MN (9).

Despite its effectiveness and safe profile (10), rituximab induces broad B cell depletion since the CD20 antigen is expressed in most B cell lineages. On the opposite, it has been estimated that autoreactive B cells in autoimmune diseases comprise only 0.1-0.5% of total circulating B cells (11). As a result, currently available B cell depleting agents are still non-specific and patients develop side effects due to impaired immune system (12, 13). Targeted suppression of autoreactive B cells remains an elusive unmet clinical need.

**Figure 1:**
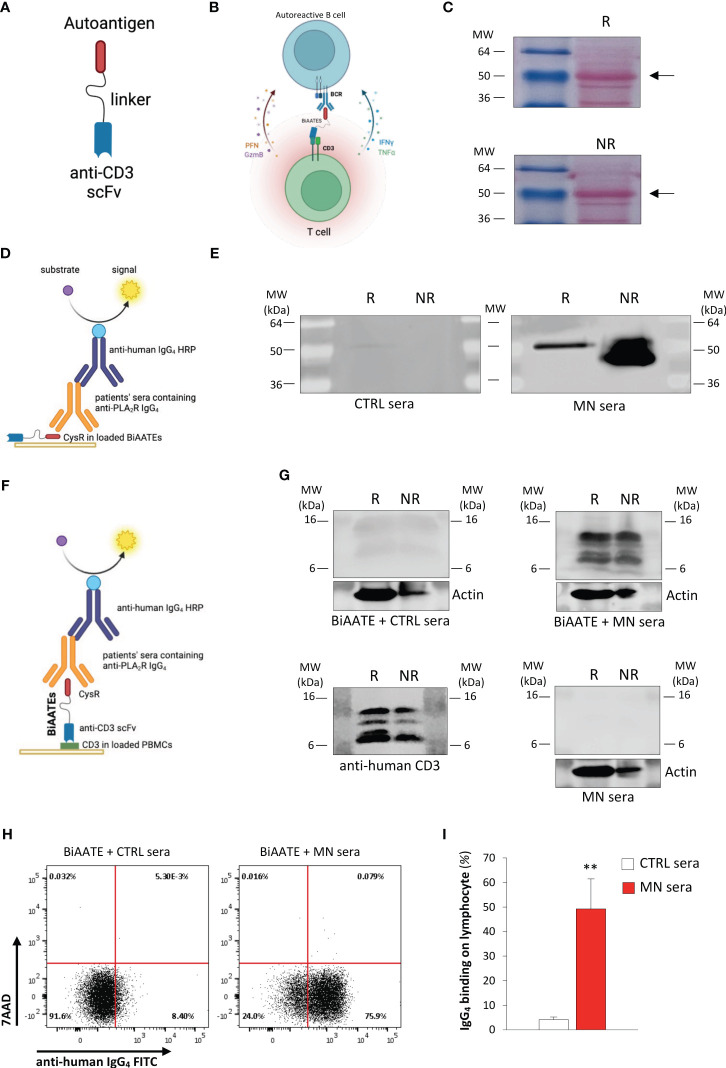
Design and characterization of the BiAATE for MN. **(A, B)** Schematic representation of the structure **(A)** and the proposed mechanism of action **(B)** of the BiAATE, a bi-specific molecule that expresses the autoantigen involved in a specific autoimmune disease linked to the single-chain variable fragment (scFv) of an anti-CD3 by a small flexible linker. As such, the BiAATE redirects the lytic activity of T cells against the surface immunoglobulin (sIg)^+^ included in the B cell receptor (BCR) of autoreactive B cells. Activation of T cells leads to the production of perforin (PFN), granzyme B (GzmB), interferon γ (IFNγ), and tumor necrosis factor α (TNFα) that induce the selective removal of autoreactive B cells. All drawings were created by using BioRender.com. **(C)** Representative ponceau red staining of 1 μg BiAATE loaded under reducing (R) and non-reducing (NR) condition. Molecular weights (MW) are reported for each Western Blot and expressed in kilo Dalton (kDa). The BiAATE is indicated by arrows and exhibited a MW of approximatively 50 kDa. **(D)** Schematic representation of the Western Blot performed to test the immunogenic properties of the CysR antigen included in the BiAATE. **(E)** Representative Western Blot of 1 μg BiAATE incubated with sera from healthy controls (CTRL sera) or MN patients (MN sera). Data derive from n=6 experiments from three independent batches of BiAATE. **(F)** Schematic representation of the Western Blot performed to test the ability of the BiAATE to simultaneously bind CD3 in T cells and the pathogenic antibodies in the MN sera. **(G)** Representative Western Blot of total peripheral blood mononuclear cell (PBMC) extracts loaded under R and NR condition and incubated with 5 μg/mL BiAATE followed by exposure to CTRL sera (BiAATE + CTRL sera) or MN sera (BiAATE + MN sera), or incubated with MN sera without prior exposure to 5 μg/mL BiAATE (MN sera). Actin was used as sample loading control. Representative western blot of CD3 expression in PBMC extracts to confirm CD3 specificity (anti-human CD3). Data derive from n=6 experiments with PBMCs isolated from 6 healthy subjects and exposed to sera from n=6 CTRL or MN patients. **(H)** Representative dot plots of anti-human IgG_4_ FITC antibody binding and 7AAD staining after gating singlet lymphocytes. PBMCs were incubated with 5 µg/mL BiAATEs in the presence of CTRL or MN sera, then with mouse anti-human IgG_4_ FITC antibody and 7AAD. **(I)** Percentage of lymphocytes bound by anti-human IgG_4_ FITC antibody after incubation with 5 µg/mL BiAATEs in the presence of CTRL or MN sera (n=6 *per* group). Data represent mean ± SEM and were analyzed using unpaired t-test. **p-value<0.01 *vs* CTRL sera.

Inspired by the clinical success of bi-specific T cell engagers (BiTEs) to treat B cell malignancies (14–16), we reasoned that autoantigen-specific B cells could be targeted by a novel class of immunotherapeutic molecules, Bi-specific AutoAntigen-T cell Engagers (BiAATEs), that express the autoantigen involved in the specific autoimmune disease and the single-chain variable fragment (scFv) of an antibody against the T cell antigen CD3 connected by a flexible linker (**Figure 1A**). With this approach, the BiAATEs physically link autoreactive B cells bearing an autoantigen-specific surface Ig^+^ to T cells, creating an immunological synapse in which activated T cells deplete only autoreactive B cells (**Figure 1B**).

In this paper, we describe the generation of a BiAATE specific for MN. This construct was evaluated for its biological activity *ex vivo* in autoreactive B cells isolated from MN patients and *in vivo* in mice transgenic for human CD3 following active immunization with PLA_2_R. The present work represents a major contribution to the field for the identification of targeted approach to selectively deplete autoreactive B cells in autoimmune diseases.

## 2 Results

### 2.1 Design and *in vitro* characterization of the BiAATE for MN

PLA_2_R is one of four members of the mannose receptor family in mammals that comprises the N-terminal cysteine-rich (CysR) domain, a single fibronectin type-2 (FnII) domain, and eight C-type lectin-like domains (CTLD1-7) (17). Available data suggest that CysR is the immunodominant domain of PLA_2_R (18). Based on these finding, we generated a DNA sequence (**Table 1**) comprising the sequence of the human CysR domain, a small flexible (GGGGS)_3_ linker, and the scFv anti-CD3 based on the sequence of anti-human CD3 monoclonal antibody OKT3.

**Table 1:** DNA sequence of the BIAATE for the treatment of MN comprising the CysR domain (red), the small flexible linker (blue), and the scFv of the anti-CD3 OKT3 (green).

DNA sequence:	aagggcatct tcgtgatcca gagcgaaagc ctgaaaaagt gcatccaggc cggaaaaagc 60 gtgctgacac tggagaactg caagcaggcc aacaagcaca tgctgtggaa atgggtgagc 120 aatcacggcc tgttcaacat cggcggcagc ggctgcctgg gcctgaactt ctccgccccc 180 gaacagcccc tgagcctgta cgagtgcgac agcaccctgg tgagcctgag atggagatgc 240 aacagaaaga tgatcacagg ccctctgcag tactccgtgc aggtggccca cgacaatacc 300 gtggtggcca gcagaaaata catccacaag tggatctctt acggcagcgg cggaggcgac 360 atctgcgagt acggcggagg cggcagcgga ggaggaggaa gtggaggcgg aggcagcgac 420 atcaagctgc agcagagcgg cgccgagctg gccagacccg gcgccagcgt gaagatgagc 480 tgcaagacca gcggctacac cttcaccaga tacaccatgc actgggtgaa gcagagaccc 540 ggccagggcc tggagtggat cggctacatc aaccccagca gaggctacac caactacaac 600 cagaagttca aggacaaggc caccctgacc accgacaaga gcagcagcac cgcctacatg 660 cagctgagca gcctgaccag cgaggacagc gccgtgtact actgcgccag atactacgac 720 gaccactact gcctggacta ctggggccag ggcaccaccc tgaccgtgag cagcgtggag 780 ggcggcagcg gcggcagcgg cggcagcggc ggcagcggcg gcgtggacga catccagctg 840 acacagagtc ccgctatcat gtctgccagc ccaggcgaaa aagtgactat gacctgcaga 900 gctagcagca gcgtgagcta catgaactgg taccagcaga agagcggcac cagccccaaa 960 agatggatct atgacacctc caaggtggcc tctggggtgc cttacagatt ctccggctcc 1020 ggcagcggca cctcctacag cctgactatc agcagcatgg aggccgagga cgccgccaca 1080 tactactgcc agcagtggag cagcaaccct ctgactttcg gcgccggcac caagctggaa 1140 ctgaag 1146

DNA was cloned in HEK293 and the corresponding protein was purified (**Table 2**). Western blot analysis was performed to validate the generated protein. Specifically, 1 μg BiAATE was loaded under reducing (R) and non-reducing (NR) conditions and transferred to nitrocellulose membranes. As shown in **Figure 1C**, ponceau red staining revealed that the BiAATE exhibited a molecular weight of approximatively 50 kDa. The immunogenicity of the CysR antigen included in BiAATE was tested by incubating the membranes with sera from healthy controls (CTRL) or MN patients, followed by anti-human IgG_4_ HRP-conjugated secondary antibody (**Figure 1D**). No reactivity was found when the BiAATE was incubated with CTRL sera (**Figure 1E**, left panel). Conversely, the CysR domain in the BiAATE was recognized by pathogenic IgG_4_ autoantibodies in MN sera (**Figure 1E**, right panel), mainly in NR conditions, confirming that the CysR included in the BiAATE is a proper conformational epitope recognized with high affinity by autoantibodies in sera of MN patients. As a positive control, a commercially available anti-PLA_2_R antibody raised against the extracellular N-terminal domain of human PLA_2_R (CysR-FnII-CTLD1-CTLD2-CTLD3) was used. This antibody recognized the CysR domain in the BiAATE only in NR conditions (**Supplementary Figure S1A**) and to a lesser extent compared to autoantibodies present in MN sera (**Figure 1E**), suggesting the predominant role of CysR as the immunogenic domain in MN.

**Table 2:** Protein sequence of the BIAATE for the treatment of MN comprising the CysR domain (red), the small flexible linker (blue), and the scFv of the anti-CD3 OKT3 (green).

Protein sequence:	Lys Gly Ile Phe Val Ile Gln Ser Glu Ser Leu Lys Lys Cys Ile Gln 1 5 10 15 Ala Gly Lys Ser Val Leu Thr Leu Glu Asn Cys Lys Gln Ala Asn Lys 20 25 30 His Met Leu Trp Lys Trp Val Ser Asn His Gly Leu Phe Asn Ile Gly 35 40 45 Gly Ser Gly Cys Leu Gly Leu Asn Phe Ser Ala Pro Glu Gln Pro Leu 50 55 60 Ser Leu Tyr Glu Cys Asp Ser Thr Leu Val Ser Leu Arg Trp Arg Cys 65 70 75 80 Asn Arg Lys Met Ile Thr Gly Pro Leu Gln Tyr Ser Val Gln Val Ala 85 90 95 His Asp Asn Thr Val Val Ala Ser Arg Lys Tyr Ile His Lys Trp Ile 100 105 110 Ser Tyr Gly Ser Gly Gly Gly Asp Ile Cys Glu Tyr Gly Gly Gly Gly 115 120 125 Ser Gly Gly Gly Gly Ser Gly Gly Gly Gly Ser Asp Ile Lys Leu Gln 130 135 140 Gln Ser Gly Ala Glu Leu Ala Arg Pro Gly Ala Ser Val Lys Met Ser 145 150 155 160 Cys Lys Thr Ser Gly Tyr Thr Phe Thr Arg Tyr Thr Met His Trp Val 165 170 175 Lys Gln Arg Pro Gly Gln Gly Leu Glu Trp Ile Gly Tyr Ile Asn Pro 180 185 190 Ser Arg Gly Tyr Thr Asn Tyr Asn Gln Lys Phe Lys Asp Lys Ala Thr 195 200 205 Leu Thr Thr Asp Lys Ser Ser Ser Thr Ala Tyr Met Gln Leu Ser Ser 210 215 220 Leu Thr Ser Glu Asp Ser Ala Val Tyr Tyr Cys Ala Arg Tyr Tyr Asp 225 230 235 240 Asp His Tyr Cys Leu Asp Tyr Trp Gly Gln Gly Thr Thr Leu Thr Val 245 250 255 Ser Ser Val Glu Gly Gly Ser Gly Gly Ser Gly Gly Ser Gly Gly Ser 260 265 270 Gly Gly Val Asp Asp Ile Gln Leu Thr Gln Ser Pro Ala Ile Met Ser 275 280 285 Ala Ser Pro Gly Glu Lys Val Thr Met Thr Cys Arg Ala Ser Ser Ser 290 295 300 Val Ser Tyr Met Asn Trp Tyr Gln Gln Lys Ser Gly Thr Ser Pro Lys 305 310 315 320 Arg Trp Ile Tyr Asp Thr Ser Lys Val Ala Ser Gly Val Pro Tyr Arg 325 330 335 Phe Ser Gly Ser Gly Ser Gly Thr Ser Tyr Ser Leu Thr Ile Ser Ser 340 345 350 Met Glu Ala Glu Asp Ala Ala Thr Tyr Tyr Cys Gln Gln Trp Ser Ser 355 360 365 Asn Pro Leu Thr Phe Gly Ala Gly Thr Lys Leu Glu Leu Lys 370 375 380 382

### 2.2 The BiAATE simultaneously binds T cells and pathogenic MN autoantibodies

We evaluated the potential of the bispecific fusion protein to simultaneously bind anti-PLA_2_R autoantibodies and CD3 *in vitro*. To this purpose, equal amounts of extracts of peripheral blood mononuclear cells (PBMCs) from healthy donors were loaded under R and NR conditions and transferred to nitrocellulose membranes. Membranes were then incubated with or without 5 μg/mL BiAATE, followed by sera from healthy CTRL or MN patients and anti-human IgG_4_ HRP-conjugated secondary antibody (**Figure 1F**). Our results showed that no reactivity was found when PBMC extracts were incubated with the BiAATEs followed by CTRL sera (**Figure 1G**, upper panel on the left). Conversely, when PBMC extracts were incubated with the BiAATE followed by MN sera, a positive signal around 16 kDa was detected, corresponding to CD3 in T lymphocytes (**Figure 1G**, upper panel on the right). The proper CD3 signal was confirmed by commercially available anti-human CD3 antibody (**Figure 1G**, lower panel on the left). No signal on CD3 was detected when PMBC extracts were exposed to MN sera in the absence of BiAATE (**Figure 1G**, lower panel on the right), confirming the specific role of the BiAATE in binding CD3 and pathogenic autoantibodies. To confirm the specific binding of BiAATE to T cells, we performed Western Blot experiments using extracts of T cells isolated from healthy donor PBMCs. In this setting, exposure of T cell extracts to 5 μg/mL BiAATE followed by CTRL sera did not result in a positive signal on CD3 (**Supplementary Figure S1B**), while incubation of T cell extracts with BiAATE and MN sera yielded a positive signal on CD3 (**Supplementary Figure S1B**).

To further corroborate *ex vivo* the potential of the bispecific fusion protein to bind simultaneously T cells and the pathogenic IgG, PBMCs from healthy donors were incubated with or without 5 μg/mL BiAATEs followed by CTRL or MN sera, FITC anti-IgG_4_ antibody and then analyzed by FACS. Singlet lymphocytes gated based on morphologic profile were plotted for FITC anti-IgG_4_ antibody and the viability marker 7AAD staining. When PBMCs were exposed to CTRL or MN sera alone, neither IgG_4_ binding nor cell death were observed, as revealed by negative staining of FITC and 7AAD (**Supplementary Figure S1C**). Conversely, a significant increase in the % of lymphocytes positive for IgG_4_ FITC antibody was observed in PBMCs incubated with the BiAATE followed by MN sera but not CTRL sera (**Figures 1H, I**). In this setting, BiAATE did not induce apoptosis, as revealed by negative staining of 7AAD (**Figure 1H**). When PBMCs were incubated with BiAATE followed by commercially available anti-human PLA_2_R mouse antibody, no IgG_4_ FITC positivity was found (**Supplementary Figure S1D**), confirming Western Blot results that anti-PLA_2_R IgG_4_ in patients’ sera have higher affinity to the CysR domain compared to commercial anti-PLA_2_R antibody.

### 2.3 The BiAATE selectively depletes anti-PLA_2_R secreting B cells isolated from MN patients

**Figure 2:**
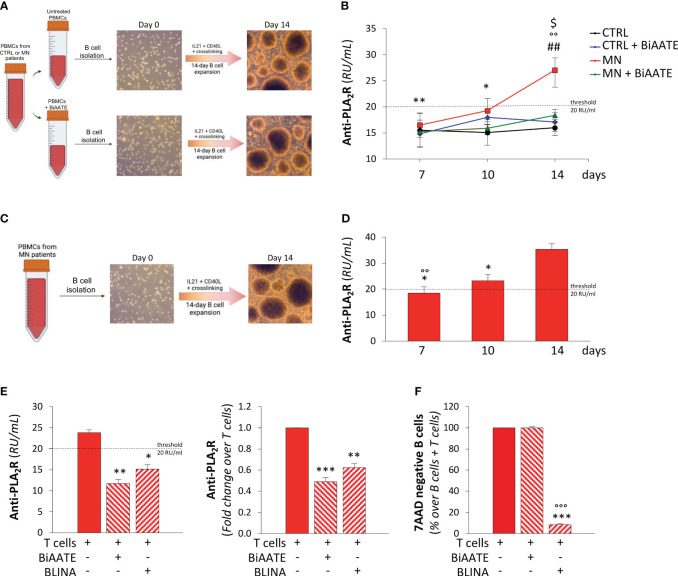
The BiAATE selectively depletes PLA_2_R-specific autoreactive B cells isolated from MN patients. **(A, B)** Schematic representation of the experimental procedure **(A)** and quantification overtime of IgG anti-PLA_2_R in the supernatants **(B)** during the expansion of B cells isolated from CTRL and MN PBMCs treated or not with 3 μg/mL BiAATE for 15 hours (n=4 *per* group). Data represent mean ± SEM and were analyzed using 2-way ANOVA corrected with Tukey *post hoc* test. *p-value<0.05, and **p-value<0.01 *vs* MN at 14 days; °°*p-value*<0.01 *vs* CTRL at the corresponding time; ^##^ *p-value*<0.01 *vs* CTRL + BiAATE at the corresponding time; and *^$^p-value*<0.05 *vs* MN+ BiAATE at the corresponding time. **(C, D)** Schematic representation of the experimental procedure **(C)** and quantification overtime of IgG anti-PLA_2_R antibody in the supernatants **(D)** during the expansion of B cells isolated from PBMCs of MN patients (n=6 *per* group). Data represent mean ± SEM and were analyzed using 1-way ANOVA corrected with Tukey *post hoc* test. *p-value<0.05 *vs* 14 days; °°*p-value*<0.01 *vs* 10 days. **(E)** Quantification of IgG anti-PLA_2_R in the supernatants of expanded B cells from MN patients treated with autologous T cells alone or in the presence of 3 μg/mL BiAATEs or 3 μg/mL Blinatumomab (BLINA) for 3 days (n=6 *per* group). Data represent mean ± SEM and were analyzed using 1-way ANOVA corrected with Tukey *post hoc* test. *p-value<0.05, **p-value<0.01, and ***p-value<0.001 *vs* T cells alone. **(F)** Percentages on singlets of viable (7AAD negative) CD3^-^CD19^+^ B cells co-cultured with autologous T cells alone or in the presence of 3 μg/mL BiAATE or 3 μg/mL BLINA for 3 days (n=6 *per* group). The percentage of viable B cells in co-culture with autologous T cells alone were taken as 100%. Data represent mean ± SEM and were analyzed using 1-way ANOVA corrected with Tukey *post hoc* test. ***p-value<0.001 *vs* T cells alone and °°°*p-value*<0.001 *vs* BIAATE.

To confirm the ability of the bispecific fusion protein to selectively deplete autoreactive B cells, we performed *ex vivo* experiments with isolated B cells (**Figure 2A**). To this aim, PBMCs from healthy CTRL or MN patients were incubated overnight with or without 3 μg/mL BiAATE. The day after, B cells were isolated and expanded as previously described (19), in order to induce B lymphocyte activation, proliferation and differentiation into antibody-secreting cells (20).

B cell phenotype was analyzed at the beginning and the end of the expansion protocol with the gating strategy depicted in **Supplementary Figures S2A–I**. First, we evaluated the changes in the expression of IgD and CD27 markers at the start and at the end of the 14-day expansion protocol of B cells from healthy CTLR and MN patients alone or treated with BiAATE (**Supplementary Figures S2B–I**). In both CTRL and MN subjects, we found a significant decrease in naïve B cell subsets paralleled by an increase in double negative B cells (**Supplementary Figure S3**), confirming that expanded B cells acquired a IgD-negative memory phenotype. Accordingly, we found a significant increase in plasma cell and plasmablast percentages during the 14-day expansion protocol in B cells from CTRL subjects (**Supplementary Figure S3**). In MN patients, plasmablast percentages did not significantly increase during the 14-day expansion (**Supplementary Figure S3**), probably due to higher numerical levels of plasmablasts at T0. Treatment with BiAATE did not impair IgD/CD27 cell subset distribution and differentiation into plasma cells and plasmablasts of B cell from either CTRL or MN patients (**Supplementary Figure S3**).

During the expansion protocol, B cell supernatants were collected at day 7, 10, and 14 and tested for anti-PLA_2_R antibody levels by antigen-coated ELISA. No anti-PLA_2_R antibody production was observed in expanded B cells isolated from both BiAATE treated and untreated PBMCs of CTRL (**Figure 2B**). Conversely, a progressive increase in anti-PLA_2_R antibody titers was found overtime during expansion of B cells isolated from untreated PBMCs of MN patients, suggesting a proper expansion of PLA_2_R-specific autoreactive B cells. When B cells were isolated from MN patients’ PBMCs treated with BiAATE, no detectable levels of anti-PLA_2_R autoantibodies were found during B cell expansion (**Figure 2B**), suggesting that incubation of BiAATE effectively redirect the cytotoxic activity of T cells in PBMCs against PLA_2_R-specific autoreactive B cells.

To confirm this key finding, an additional protocol was set up to test depletion of autoreactive anti-PLA_2_R B cells in MN patients (**Figure 2C**). In this setting, B cells were isolated from untreated PBMCs of MN patients and expanded for 14 days with the protocol described above. As shown in **Figure 2D**, we confirmed the progressive increase in anti-PLA_2_R antibody titers in supernatants during the expansion of B cells. At 14 days, expanded B cells were incubated with autologous T cells isolated from the same MN patient alone or in the presence of 3 μg/mL BiAATE or 3 μg/mL blinatumomab (BLINA), an anti-CD3 and anti-CD19 bispecific antibody approved for the treatment of refractory B cell precursor acute lymphoblastic leukemia. After 3 days of treatment, analysis of supernatants revealed that, compared to untreated cells, the BiAATE induced a strong reduction of anti-PLA_2_R antibody levels that declined under the threshold level of the assay (**Figure 2E**). A similar effect in reducing anti-PLA_2_R antibody levels was achieved by BLINA (**Figure 2E**). At the end of lymphocyte co-cultures, the overall cell vitality was similar in the three experimental conditions (**Supplementary Figure S4A**). By gating viable cells for the expression of CD3 and CD19, the percentage of live B cells was marginally affected in the presence of BiAATE, while it was markedly decreased by BLINA (**Supplementary Figure S4B**). Similar results were obtained when gating viable cells for the expression of CD3 and CD20 (**Supplementary Figure S4C**). Quantification of live B cells in co-culture experiments with lymphocytes from 6 distinct MN patients revealed that BiAATE had a negligible effect on B cell death, while BLINA elicited a marked 90% decrease in B cell viability (**Figure 2F**).

### 2.4 The BiAATE reduces anti-PLA_2_R levels in immunized mice transgenic for human CD3

To analyze the full spectrum of the therapeutic potential of the bispecific fusion protein, we pursued on testing the BiAATE *in vivo*. In order to identify the most suitable animal model, we performed a Western Blot analysis to test the reactivity of the BiAATE against individual human CD3 subunits (21). As shown in **Supplementary Figure S1B**, when recombinant CD3δ, CD3ϵ, and CD3γ proteins were exposed to 5 μg/mL BiAATE followed by MN sera, no IgG_4_ signal was detected on individual CD3 subunits, whereas IgG_4_ signal was found on the fully assembled CD3 in extracts from PBMCs and isolated T cells from healthy donors. These data suggest that the anti-CD3 scFv antibody in the BiAATE does not recognize individual CD3 subunits but requires an intact human CD3 for a proper recognition.

**Figure 3:**
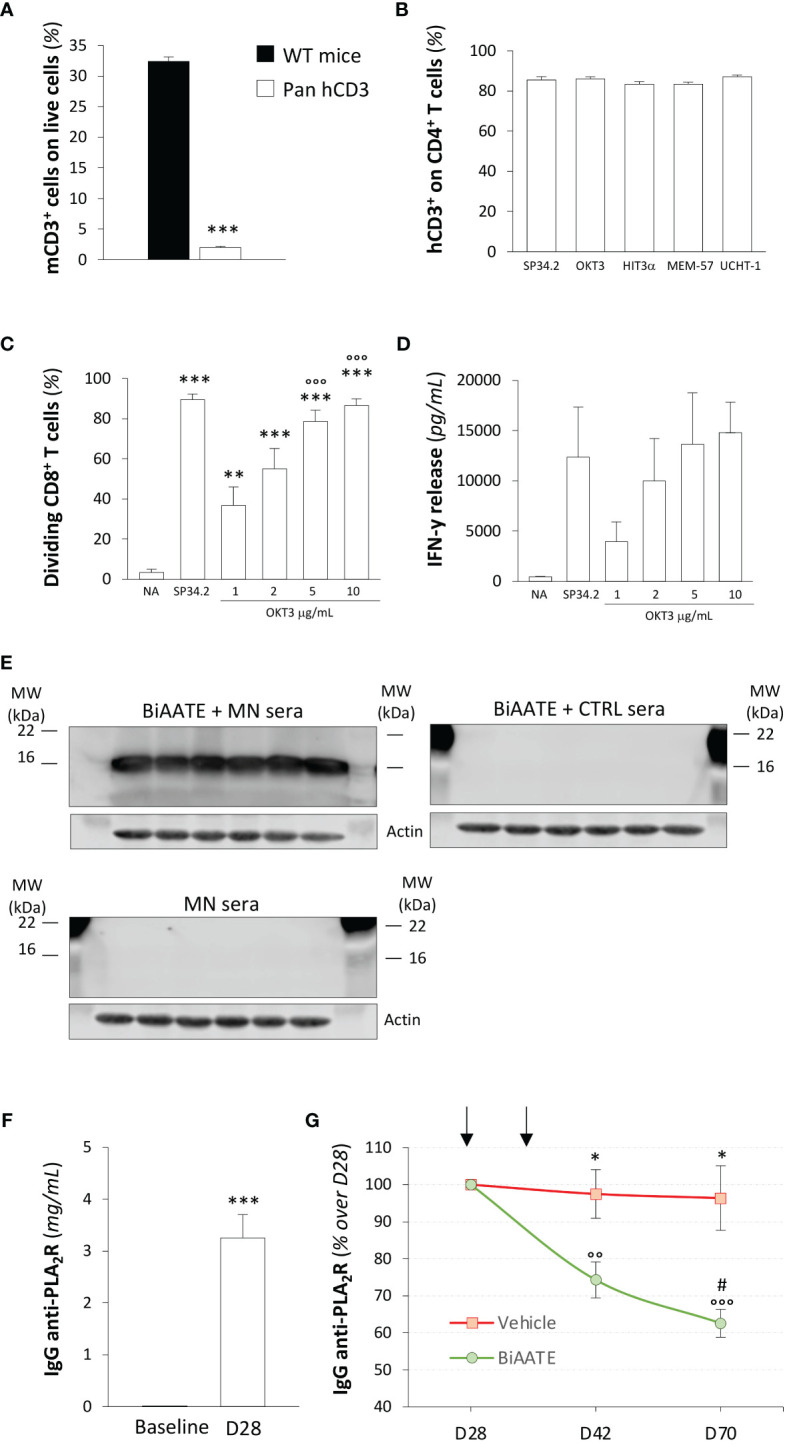
Human CD3 is expressed and functional on Pan hCD3 mice, and the BiAATE reduces PLA_2_R antibody titer in these mice following active immunization with PLA_2_R. **(A)** Expression of mouse CD3 in splenocytes from Pan hCD3 and WT mice (n=3) determined flow cytometry. Data represent mean ± SEM and were analyzed using unpaired t-test. ***p-value<0.001 *vs* WT mice. **(B)** Binding of different antibody clones of human anti-CD3 (SP34.2, OKT3, HIT3α, MEM-57, UCHT-1) to CD4^+^ T cells from Pan hCD3 mice (n=3) determined by flow cytometry. **(C, D)** Percentage of dividing CD8^+^ T cells **(C)** and IFN-γ release **(D)** determined by flow cytometry and ELISA, respectively, in unstimulated (NA) T cells isolated from Pan hCD3 or in response to coated human anti-CD3 (OKT3) activation for 3 days at different concentration (n=6 *per* group). Human anti-CD3 antibody clone SP34.2 (5 µg/mL) was used as a positive control. Data represent mean ± SEM and were analyzed using 1-way ANOVA corrected with Tukey *post hoc* test. **p-value<0.01, and ***p-value<0.001 *vs* NA; °°°*p-value*<0.001 *vs* OKT3 1 μg/mL. **(E)** Representative Western Blot of MN patients’ IgG_4_ binding to CD3 in total splenocyte extracts from hCD3 mice (n=6) incubated with (upper panel on the left) or without (lower panel on the left) 5 μg/mL BiAATE followed by MN sera. The right panel shows representative Western Blot of IgG_4_ binding to CD3 in total splenocyte extracts from hCD3 mice (n=6) incubated with 5 μg/mL BiAATE followed by incubation with CTRL sera. Actin was used as sample loading control. MW are reported in each Western Blot and expressed in kDa. **(F)** Quantification of IgG anti-PLA_2_R (mg/mL) in the sera of pan hCD3 mice (n=18) immunized with two doses of 50 μg PLA_2_R antigen. Data represent mean ± SEM and were analyzed with unpaired t-test. ***p-value<0.001 *vs* Baseline. **(G)** Quantification overtime of IgG anti-PLA_2_R expressed as % changes over D28 in the sera of immunized pan hCD3 mice (n=9 *per* group) treated at D28 and D35 (black arrows) with vehicle or 1 mg/kg BiAATE. Data represent mean ± SEM and were analyzed with 2-way ANOVA corrected with Šídák’s multiple comparisons test. *p-value<0.05 *vs* BiAATE at the corresponding time; °°*p-value*<0.01, and °°°*p-value*<0.001 *vs* BiAATE at D28; and ^#^p-value<0.05 *vs* BiAATE at D42.

In light of this evidence, we elected to use a transgenic mouse model expressing the human CD3εγδ subunits (Pan hCD3) to maximize the binding activity of the anti-CD3 scFv antibody in the BiAATE. As expected, Pan hCD3 mice lacked the expression of mouse CD3 on splenocytes compared to WT mice (**Figure 3A**, **Supplementary Figure S5A**), while they expressed human CD3 as detected by staining with different anti-human CD3 antibody clones, including SP34.2, OKT3, HIT3α, MEM-57 and UCHT-1 (**Figure 3B**, **Supplementary Figure S5B**). In splenocytes from Pan hCD3 mice, stimulation with OKT3 induced CD8 T cell proliferation in a concentration-dependent manner (**Figure 3C**, **Supplementary Figure S5C**), which was paralleled by IFN-γ production (**Figure 3D**), suggesting that the complex CD3-TCR was stable and functional.

Given that CD3 plays a key role in thymocyte development (22), we investigated whether the humanization of CD3 impaired this process. Total number of thymocytes was similar in Pan hCD3 and WT mice (**Supplementary Figure S6A**). Frequency of double negative, double positive, single positive CD4 and single positive CD8 subpopulations in thymus from Pan hCD3 were unchanged compared to WT mice (**Supplementary Figure S6B**), suggesting that the humanization of CD3 did not affect the thymocytes maturation process. Moreover, splenocyte number (**Supplementary Figure S6A**) and the T cell distribution in the spleen was not altered in Pan hCD3, as the frequency of TCRβ^+^ and TCRγδ^+^ or CD8, CD4 and Treg subpopulations did not show any major differences compared with WT mice (**Supplementary Figure S6C, D**).

To validate the binding of the BiAATE to T cells from Pan hCD3 mice, we performed Western Blot experiments. When splenocyte extracts were exposed to the BiAATE followed by MN patients’ sera, we found a significant binding of MN patients’ IgG_4_ to CD3 (**Figure 3E**, upper panel on the left), suggesting a proper reactivity of the BiAATE against humanized CD3 in transgenic mice. Conversely, no signal on CD3 was found when the BiAATE was omitted before the incubation with MN patients’ sera (**Figure 3E**, lower panel on the left), confirming the specificity of the BiAATE. No IgG_4_ reactivity on CD3 was found when splenocyte extracts were exposed to the BiAATE followed by CTRL sera (**Figure 3E**, right panel).

Based on these results, pan hCD3 mice were used for active immunization with PLA_2_R. To this aim, we generated and purified full-length PLA_2_R construct and tested it by Western Blot. As shown in **Supplementary Figure S6E**, the PLA_2_R construct was highly reactive following exposure to MN patients’ sera only in NR conditions, suggesting that the generated protein has proper conformational epitopes that replicate antigen immunogenicity in MN. At baseline, all mice were negative for anti-PLA_2_R antibodies (**Figure 3F**) and received a prime-boost intramuscular immunization with 50 μg PLA_2_R, each administered two weeks apart. Fourteen days after the second dose, all mice developed a strong humoral response against PLA_2_R (**Figure 3F**), confirming that they were fully immunocompetent. On day 28 (D28), mice were assigned to receive an intravenous injection of vehicle or 1 mg/kg BiAATE following 1:1 block randomization for the levels of anti-PLA_2_R antibodies (IgG anti-PLA_2_R: vehicle 3.1 ± 0.7 mg/mL *vs* BiAATE 3.4 ± 0.6 mg/mL; p=0.7253). A subsequent injection of vehicle or BiAATE was performed on D35. As shown in **Figure 3G**, pan hCD3 mice that received vehicle exhibited a sustained humoral response up to D70, whereas mice receiving the BiAATE showed a significant overtime decrease in the levels of anti-PLA_2_R antibodies.

## 3 Discussion

In this study, we designed and tested the BiAATEs, the first-in-class immunotherapeutic molecules to achieve effective depletion of autoreactive B cells in autoimmune diseases. To this purpose, we focused on MN, an autoimmune disease of the kidney, in which PLA_2_R is the main autoantigen.

To design a BiAATE for MN, we took advantage of a recent evidence showing that anti-PLA_2_R autoantibodies circulating in the blood of patients with MN essentially bind to a dominant epitope located within a 31-amino acid peptide of PLA_2_R within the CysR domain (18). That antibodies against the CysR domain are pathogenic derives from our data in 113 patients with MN, where we document that depletion of anti-CysR antibodies, rather than antibodies against other domains, was associated with a better outcome following rituximab treatment (23). Based on these findings, we generated a BiAATE comprising the full-length CysR domain, corresponding to the residue 38-161 of the PLA_2_R protein. The CysR domain was connected to the scFv of the anti-human CD3 monoclonal antibody by a small linker to allow an adequate flexibility of the molecule. The anti-CD3 scFv was derived from the sequence of OKT3, the first FDA-approved anti-human CD3 monoclonal antibody for treating acute rejection in transplant recipients (24).

Here, we provided *in vitro* and *ex vivo* evidence that the BiAATE is endowed with a proper flexibility to simultaneously bind pathogenic IgG_4_ autoantibodies in MN sera to CD3 in T cells isolated from healthy donors. Thus, the BiAATE has the potential to create an immunological synapse between autoreactive B cells bearing a PLA_2_R-specific surface Ig^+^ and T cells. The formation of this immunological synapse crucially depends on the distance between the two engaged immune cells. Finding that the 50 kDa molecular weight of BiAATE is similar to that of blinatumomab (54 kDa) indicates that the BiAATE had a steric bulk likely permissive for this immunological synapse to form. In this setting, we documented that the BiAATE is indeed effective *ex vivo* in redirecting the cytotoxic activity of T cells against PLA_2_R-specific autoreactive B cells taken from patients with MN in a rather selective fashion, fully sparing normal B cells. This property would represent a peculiar advantage of the BiAATE over the currently available immunotherapies, including blinatumomab, that unselectively depletes most of circulating B cells.

A further advantage of the BiAATE is the potential of targeting autoantigen-specific Ig^+^ autoreactive B cells, including plasma cells. Plasma cells lack the B cell antigens – namely CD19 and CD20 –and are therefore resistant to currently available bispecific and monoclonal antibodies. This is a critical issue considering that the percentages of circulating plasma cells are correlated with serum anti-PLA_2_R antibody levels and clinical signs of MN in patients (25). Furthermore, a unique population of long-lived and mature plasma cells expressing IgG^+^ but lacking CD19 has been identified in the bone marrow and in chronically inflamed synovial tissue of patients with autoimmune rheumatoid arthritis where these cells secrete autoreactive antibodies (26). This stable plasma cell population accounts for long-term autoantibody production in spite of B cell depletion (26), but could be efficiently targeted by the BiAATE due to their expression of autoantigen-specific IgG.

Recently, an innovative cellular immunotherapy has been proposed for antigen-specific B cell depletion in the autoimmune disease pemphigus vulgaris (PV). This approach is the engineering of T cells with a chimeric autoantibody receptor expressing the PV autoantigen fused to CD137-CD3ζ signaling domain, thus conferring to T cells the ability to kill B cell specific for the PV autoantigens (27, 28). Despite promising, T cell manufacturing requires several complex, expensive and time-consuming steps such as leukapheresis, *ex vivo* T cell transduction, expansion of engineered T cells and patient transfusion. At variance, the BiAATEs are recombinant proteins that can be manufactured in large quantities without inter-patient variability. While scalability remains to be explored, the versatility of BiAATEs could make it a valuable off-the-shelf compound that can be rapidly used once the clinical indication has been determined. Lastly, it is worth emphasizing that adoptive T cell transfer necessitates severe preconditioning regimen with lymphodepleting chemotherapy to facilitate the engraftment of engineered T cells (29), imposing a significant strain on patients’ immune systems. Conversely, the utilization of BiAATEs obviates the necessity for such preconditioning, thereby mitigating the potential impact on patient well-being.

To offer *in vivo* evidence of the potential effect of the recombinant protein as an actual therapeutic tool, we tested the BiAATE in humanized CD3 mice. This condition was necessary given the fact that the ectodomains of CD3 subunits are poorly conserved between mice and humans (30). In addition, previous studies showed that the proper formation of CD3ϵγ heterodimer complexes is an essential prerequisite for the therapeutic binding of anti-human CD3 monoclonal antibodies, particularly the OKT3 clone (31). Considering that the anti-human CD3 scFv antibody included in the BiAATE was based on the sequence of OKT3, we used a transgenic mouse model in which all the three subunits of the murine CD3 – ϵ, δ, and γ – are replaced by their human counterparts, allowing us to maximize the binding activity of the BiAATE to humanized murine T cells. These mice exhibited a full human CD3 in T lymphocytes, which were susceptible to OKT3 activation and were effectively recognized by the BiAATE *ex vivo*. Additionally, the genetic manipulation did not alter neither the thymocyte maturation process, nor CD3-TCR stability and function. As a result, these mice were fully immunocompetent, as revealed by robust murine humoral response following active immunization with PLA_2_R antigen. Treatment of immunized pan hCD3 mice with the BiAATE effectively reduced antibody titer by almost the 40% at the end of the experimental period. Finding that antibody reduction persisted over a month since the last BiAATE injection may suggest that the effect on circulating antibodies was attributable to the reduction of anti-PLA_2_R B cells. These data hint that such a strategy may be valuable in redirecting the killing activity of T cells against anti-PLA_2_R secreting B cells in the context of adaptive immune response *in vivo*. To date a suitable animal model for human PLA_2_R-related MN is not available (32), ruling out the possibility to target PLA_2_R-secreting B cells to definitively prove the robustness of our approach in reducing the clinical manifestation of MN.

In conclusion, the studies described in this paper embody a proof-of-concept that BiAATEs may provide an effective and versatile platform for the targeting of autoreactive B cells in autoimmune diseases. Should this approach be confirmed for other autoimmune diseases, the BiAATEs will be fertile ground for the future production and adaption to virtually all antibody-mediated autoimmune disease for which the pathogenic autoantigen is known, leading to a paradigm shift in the treatment of these diseases.

## 4 Materials and methods

### 4.1 Ethics statement

The BICONNECTED study, involving human subjects, was reviewed and approved by the Ethical Committee of the Azienda Socio Sanitaria Territoriale Papa Giovanni XXIII, Bergamo, Italy (approval number REG. SPERIM. N. 330/20). The study conforms to the principles of the Helsinki Declaration and written informed consent was obtained from all enrolled subjects. Study participation was voluntary. No potentially identifiable human images or data are presented in this study. Baseline characteristics of healthy subjects and MN patients are summarized in **Supplementary Tables S1**, **S2**, respectively.

### 4.2 Animal experiments

Generation of a Pan-CD3 humanized mouse model was designed and developed by genOway (Lyon, France). Mouse Cd3γ, Cd3δ and Cd3ε genes were humanized subsequently by gene targeting in C57BL/6N embryonic stem (ES) cells.

### 4.3 Construction of targeting vectors for homologous recombination in embryonic stem cells

The homology arms were isolated by cloning from C57BL/6N mouse genomic DNA. Three different targeting vectors were generated to humanize the three different genes, part of the Cd3 locus: a first vector composed of a CD3γ cDNA upstream of a lox2272-flanked neomycin cassette has been inserted in frame with Cd3γ exon 2; a second vector composed of a CD3δ cDNA upstream of an FRT-flanked hygromycin cassette has been inserted in frame with murine Cd3δ exon 2; a third vector composed of a CD3ε complete cDNA upstream of a loxP-flanked puromycin cassette has been inserted in frame with murine Cd3ε exon 3. All targeting were performed in cis. The insertion of the human sequences under the control of the mouse promoters and regularity regions prevents the production of the three endogenous mouse Cd3γ, Cd3δ and Cd3ε proteins. The integrity of the targeting vectors was assessed by full sequencing.

### 4.4 Homologous recombination in embryonic stem cells

The three linearized targeting vectors were sequentially transfected into C57BL/6N ES cells according to genOway’s standard electroporation procedures and using the appropriate antibiotic selection. Antibiotic resistant ES cell clones were subsequently validated by PCR, using primers hybridizing within and outside the targeted loci, and the whole recombined loci were sequenced to confirm the absence of genetic alteration.

### 4.5 Generation of the Pan CD3 humanized mouse line (Pan hCD3)

Recombined ES cell clones with cis humanization Cd3γ, Cd3δ and Cd3ε were microinjected into mouse blastocysts, which gave rise to male chimeras with a significant ES cell contribution. Breeding was established with C57BL/6N mice expressing Flp and Cre-recombinase, to produce the Pan CD3 humanized line devoid of the resistance cassettes. Heterozygous mice were genotyped by PCR, and further validated by full sequencing of the targeted locus, including the homology arms upstream and downstream regions. Homozygous mice were produced by heterozygous interbreeding and were obtained in accordance with the expected mendelian ratio.

### 4.6 Characterization of Pan hCD3 mice

Mice were anesthetized with isoflurane followed by final sacrifice using cervical dissociation. Splenocytes and thymus were harvested from 6- to 12-week-old female and male Pan hCD3 mice, and digested using spleen dissociation kit and GentleMACS Octo Dissociator with Heaters (Miltenyi Biotec) per manufacturer’s instructions or mechanical digestion, respectively. Undigested tissues and debris were removed by filtering the cellular solution through a 70 μM filter. Total number of cells was determined using and automated cell counter (Luna-FL™, Logos Biosystems). Cells were resuspended in FACS buffer (PBS 1X, 3% FBS, 2mM EDTA), labeled with antibody cocktails and incubated for 30 min at 4°C in the dark. Expression of human CD3 on CD4^+^ cells was evaluated by flow cytometry using different clones of anti-human CD3: SP34.2, MEM-57 (BD Biosciences), UCHT1, HIT3a, and OKT3 (BioLegend). Expression of mouse CD3 on live splenocytes was evaluated using clone 145-2C11 (BioLegend). Other antibodies (CD4 – BD Biosciences, CD8a, FoxP3 – eBioscience, TCRγδ, TCRβ, CD25 – BioLegend) were used as per manufacturer’s instructions to identify cell populations. Cells were then washed in FACS Buffer before flow cytometry acquisition (Attune NxT, ThermoFisher). Intracellular staining, when necessary, was performed using Perm/Fix buffer (eBioscience) and antibody was incubated for 1h at 4°C in the dark. Data analysis was performed using FlowJo (BD Biosciences) software.

Human anti-CD3-mediated activation of T cells was evaluated on T cells isolated from splenocytes (Pan T cell isolation kit, mouse – Miltenyi Biotec) following manufacturer’s instructions. Isolated T cells were stained with CellTrace™ Violet Cell Proliferation as recommended by manufacturer (Invitrogen) and activated with coated human anti-CD3 SP34.2 (5µg/mL, BD Biosciences) or different concentrations of OKT3 (BioLegend) for 3 days, at 37°C. Supernatant was harvested for measurement of IFN-γ production by ELISA (Invitrogen) and cell proliferation was determined by flow cytometry. For that, cells were stained with CD4, CD19 and CD8, and then washed in FACS Buffer before flow cytometry acquisition (Attune NxT, ThermoFisher).

### 4.7 *In vivo* immunization studies

*In vivo* immunization studies were performed at Istituto di Ricerche Farmacologiche Mario Negri IRCCS. All procedures involving animals were performed in accordance with institutional guidelines in compliance with national (D.L.n.26, March 4, 2014), and international laws and policies (directive 2010/63/EU on the protection of animals used for scientific purposes). This study was approved by the Institutional Animal Care and Use Committees of Istituto di Ricerche Farmacologiche Mario Negri IRCCS and by the Italian Ministry of Health (approval number 438/2021-PR).

Eight-week-old female (*n*=9) and male (*n*=9) Pan hCD3 mice were maintained in a specific pathogen-free facility at a constant temperature with a 12:12-h light/dark cycle and free access to a diet and water. At day 0, all mice received a prime-boost intramuscular immunization according to standard protocol (33). Briefly, mice receive a first intramuscular injection of 50 μg phospholipase A_2_ receptor (PLA_2_R) C-terminal uncleavable protein in complete Freund′s Adjuvant (Sigma Aldrich, F5881). At day 14, mice received a second intramuscular injection of 50 μg PLA_2_R C-terminal uncleavable protein in incomplete Freund′s Adjuvant (Sigma Aldrich, F5506). At day 28, after 1:1 block randomization for the levels of anti-PLA_2_R antibodies developed two weeks from the second dose, mice were assigned to receive an i.v. injection of vehicle or 1 mg/kg BiAATE. A second i.v. injection of vehicle or BiAATEs was performed on day 35. Serum samples were collected and mice were followed until day 70. No inclusion or exclusion parameters were used in our studies. Investigators were blinded to treatments and no subjective assessments were made. This study was carried out in compliance with the ARRIVE guidelines (34).

### 4.8 PLA_2_R plasmid generation, recombinant expression and purification

The DNA sequence encoding for the PLA_2_R ectodomain (NCBI Reference Sequence: NM_007366.5) was synthesized and transferred into pUPE vectors (U-Protein Express B.V.) for secreted expression of non-cleavable C-terminal His-tagged variant of the recombinant protein product. The plasmid was used to transfect human embryonic kidney (HEK293-F) cells, as previously described (35). Briefly, cells were transfected at a density of 10^6^ cells/mL, with 1 µg of DNAx10^6^ cells and 5 µg of polyethylenimine. Four hours post-transfection, 0.6% of Primatone RL was added to the cell suspension. After 6 days, cells were harvested by centrifugation. For PLA_2_R purification, the clarified medium was filtered with a 0.8 µ filter (Sartorius) and buffer adjusted using buffer A (50 mM NaH_2_PO_4_, 500 mM NaCl, pH 8.0). For purification, the sample was loaded on a HisTrap Excel 5 mL affinity column (Cytiva) mounted on an NGC Chromatography System (Bio-Rad). Elution was carried out by applying a 0-500 mM imidazole gradient in buffer A. Eluted protein fractions were pooled and concentrated with a 100 kDa MWCO Amicon Ultra centrifugal filter (Merck) at 4,000 *x* g at 18°C. To remove excess imidazole from the sample, buffer exchange was performed by applying serial dilutions with PBS during sample concentration. All samples were concentrated to 1 mg/mL, flash-frozen in liquid nitrogen and kept at -80°C until usage.

### 4.9 BiAATE plasmid generation, recombinant expression and purification

The full DNA sequences comprising the sequence of the human CysR domain (NCBI Reference Sequence: NM_007366.5), a small flexible (GGGGS)_3_ linker, and the scFv anti-CD3 based on the sequence of anti-human CD3 monoclonal antibody OKT3 (PubChem SID: 472422762). The DNA was then cloned into a pcDNA3-based expression vector (V79020, Invitrogen) using standard molecular cloning techniques with optimized codons for transfection in HEK293-F cells. Cells were transiently transfected with this vector using Lipofectamine, ultimately expressing the fusion peptide composed by the CysR domain, a polypeptide (GGGGS)_3_ linker, and the scFv anti-CD3 antibody. The BiAATE also included a C-terminal 6xHis tag. Following a 5-day expression period, nickel columns (Bio-Rad) were used for immobilized metal affinity chromatography (IMAC) for BiAATE purification. The purified protein was then subjected to ultrafiltration and 0.2 μm sterile filtration to get the bulk of high purity. The Limulus Amebocyte Lysate (LAL) test was used for detecting bacterial toxins the final high purity product. The protein concentration was calculated with nanodrop with the extinction coefficient of 2.177. All samples were concentrated to 1 mg/mL, flash-frozen in liquid nitrogen and kept at -80°C until usage. Three independent batches of BiAATEs were tested.

### 4.10 Peripheral blood mononuclear cell and T cell isolation

Peripheral blood mononuclear cells (PBMCs) were isolated from peripheral blood by using Ficoll-Paque density centrifugation (Histopaque, 1077 from Sigma Aldrich), as we previously described (36). In selected experiments, T cells were isolated from PBMCs of healthy donors by MACS (untouched pan human T Cell isolation kit, Miltenyi, 130-096-535). Isolated PBMCs and T cells were counted, cell viability was assessed by trypan blue staining (Invitrogen) and used for subsequent experiments.

### 4.11 Protein extraction

For western blot analysis, 10x10^6^ PBMCs and 2x10^6^ isolated T cells were homogenized in 300 μl of CelLytic M (Sigma-Aldrich, C2978) supplemented with protease inhibitor cocktail (Sigma-Aldrich, P8340) containing 104 mM AEBSF at, 80 μM Aprotinin, 4 mM Bestatin, 1.4 mM E-64, 2 mM Leupeptin and 1.5 mM Pepstatin A. After sonication, cell disruption was completed by using a blunt-ended needle and a syringe and the sample lysates were then centrifuged at 16,000*x*g for 10 minutes at 4°C to remove detergent-insoluble material. Total protein concentration was determined using DC™ assay (Bio-Rad Laboratories, 5000112).

### 4.12 Western blot analysis

Western blot analysis was performed in order to validate the generated BiAATE, as we previously described (37). Briefly, 1 μg of the BiAATE was loaded on 12% SDS-PAGE under reducing and non-reducing conditions and transferred to 0.2 µm nitrocellulose membranes (Bio-Rad Laboratories, 1704159). In selected experiments, 2 µg of CD3δ (Abnova, H00000915), CD3ϵ (Abnova, H00000916), and CD3γ (Abnova, H00000917) were used. Ponceau S solution (Sigma-Aldrich, P7170) and SeeBlue™ Plus2 Pre-Stained Protein Standard (ThermoFisher, Invitrogen, LC5925) were used to identify molecular weights. After blocking with 5% bovine serum albumin (BSA) in Tris-buffered saline (TBS) supplemented with 0.1% Tween-20, membranes were incubated with healthy controls’ sera (1:10), MN patients’ sera (1:10), or with a mouse anti-human PLA_2_R antibody (1:1,000; abcam, ab211490).

For Western Blot experiments with PBMCs and T cells from human samples or hCD3 splenocytes, equal amounts (30 μg) of total PBMC, isolated T cell and splenocyte extracts were loaded on 15% SDS-PAGE under reducing and non-reducing conditions and transferred to 0.2 µm nitrocellulose membranes. After blocking, membranes were incubated with or without 5 μg/mL BiAATE followed by healthy controls’ sera (1:10), MN patients’ sera (1:10). As a control, a commercially available mouse anti-human CD3 antibody (1:1,000; Biocare Medical, PA0553 clone LN10) was used. Rabbit anti-actin (Sigma Aldrich, A5060; 1:3,000) was used as sample-loading.

The detection was performed using a mouse anti-human IgG_4_ HRP-conjugated secondary antibody for samples incubated with patients’ or controls’ sera (1:10,000; Invitrogen, A-10654) and with a goat anti-mouse IgG HRP-conjugated secondary antibody (1:20,000; Invitrogen, 31430) for samples exposed to a commercially available anti-PLA_2_R or anti-CD3 antibody. The signals were visualized on an Odyssey^®^FC Imaging System (LiCor) with SuperSignal™ West Pico PLUS Chemiluminescent Substrate (ThermoFisher, 34580) and acquired by using the Image Studio Lite 5.0 (LiCor) software. For actin, the signals were visualized by infrared (IR) fluorescence using a secondary goat anti-rabbit IRDye 680LT antibody (LiCor, FE3680210; 1:1,000).

### 4.13 FACS analysis in peripheral blood mononuclear cells

To evaluate *ex vivo* the potential of the bispecific fusion protein to bind simultaneously CD3 on T cells and the pathogenic IgG_4_ autoantibodies, 1x10^6^ PBMCs were incubated with 5 μg/mL BiAATE for 1 hour, followed by healthy controls’ sera (1:10), MN patients’ sera (1:10), or commercially available mouse anti-human PLA_2_R antibody (1 μg/mL; abcam, ab211490) for 1 additional hour and then with mouse anti-human IgG_4_ FITC (Sigma Aldrich, F9890) or a BUV395 rat anti-Mouse IgG2b (BD Biosciences, 743180) secondary antibody in the presence of the viability dye 7-Amino-Actinomycin D (7AAD, BD Bioscience, 559925). Singlet lymphocytes were gated based on their morphologic profile and plotted for the binding of FITC anti-human IgG4 or BUV395 anti-Mouse IgG2b and 7AAD to identify live 7AAD negative cells. Analysis was performed by FACS Fortessa X20 (BD) and analyzed with FlowJo Software.

### 4.14 B cell isolation, expansion and treatments

In the experimental setting of **Figure 2A**, 20x10^6^ PBMCs of MN patients were treated overnight with or without 3 μg/mL BiAATES. The subsequent day, B cells were isolated from untreated or treated PBMCs by magnetic beads (untouched human B Cell isolation kit II, Miltenyi; 130-091-151). Following isolation, B cells were seeded at a final density of 0.15×10^6^ cells/mL in StemMACS HSC Expansion Media XF (B Cell Expansion Kit, human, Miltenyi, 130-106-196) supplemented with 5% AB serum, CD40 Cross-linker (19), and IL-21 25 ng/mL (20). Under these experimental conditions, B cell expansion of up to 10-fold can be achieved. At day 7, 10, and 14, B cells were harvested, counted, and reseeded 0.15×10^6^ cells/mL in fresh medium. At each time point, supernatants were collected for anti-PLA_2_R antibody levels evaluation by enzyme-linked immunosorbent assay (ELISA).

In the experimental setting of **Figure 2C, B**, cells were isolated from untreated 20x10^6^ PBMCs from MN patients and expanded as described above. At each time point, supernatants were collected for anti-PLA_2_R antibody levels evaluation by ELISA. At day 14, expanded B cells were incubated at a 1:2 ratio with autologous T cells isolated from PBMCs of the same MN patient by magnetic beds (pan human T Cell isolation kit, Miltenyi, 130-096-535) alone or in the presence of 3 μg/mL BiAATES or 3 μg/mL Blinatumomab (BPS bioscience, BPS-100836). After three days, supernatants were collected for anti-PLA_2_R analysis by ELISA. B cells viability was assessed by 7AAD (BD Bioscience) and expressed as the % of 7AAD negative B cells compared to untreated B and T cell co-cultures, taken as 100%.

### 4.15 FACS analysis of B cell phenotype

Before and at the end of expansion protocols, B cells were incubated with the following monoclonal antibodies: CD3 BV510 (Clone Hit3a), CD19 PE-Cy7 (Clone HIB19), CD20 APC-H7 (Clone 2H7), IgD FITC (Clone IA6-2), CD27 APC-R700 (Clone M-T271), CD38 APC (Clone HIT2), CD138 BUV395 (Clone MI15). Naïve (IgD^+^CD27^-^), unswitched (IgD^+^CD27^+^), switched (IgD^-^CD27^+^) and double negative (IgD^-^CD27^-^) B cells were evaluated and expressed as percentages of viable CD19^+^CD20^+^B cells. Plasmablasts and plasma cells were identified as CD138^-^CD38^+^CD27^+^ cells and CD138^+^CD38^+^ cells, respectively and expressed as percentages of viable CD19^+^CD20^-^ B cells. Analysis was performed by FACS Fortessa X20 (BD) and analyzed with FlowJo Software.

### 4.16 Anti-PLA_2_R human IgG enzyme-linked immunosorbent assay

The B cell supernatants were tested for anti-PLA_2_R human IgG antibody levels by antigen-coated ELISA (Euroimmun, EA1254-9601G), according to the manufacturer’s instructions. Measurement of O.D. was performed on the multimode microplate reader TECAN Infinite M200® PRO at 450 nm with a reference wavelength of 650 nm. Data were obtained with a best-fit standard curve determined by regression analysis using a four-parameter logistic curve fit (4-PL) and expressed as RU/mL.

### 4.17 Anti-PLA_2_R mouse IgG enzyme-linked immunosorbent assay

For analysis of anti-PLA_2_R mouse IgG, we set up an in-house ELISA with Starter Accessory Kit (Bethyl Laboratories, E101). Briefly, microtiter wells were coated overnight with 2 μg/mL PLA_2_R C-Terminal uncleavable protein in 0.1 M Carbonate buffer. Microtiter plates were then washed and incubated with blocking buffer (50 mM Tris buffered saline, pH 8.0, 1% BSA) for 30 minutes at room temperature. Serum samples were diluted 1:100,000 in sample diluent and incubated on microtiter wells for 1 hour at 37°C. Anti-PLA_2_R mouse IgG detection was performed by incubation with goat anti-mouse IgG HRP-conjugated secondary antibody (1:20,000; Invitrogen, 31430), followed by Enzyme Substrate. Measurement of O.D. was performed on the multimode microplate reader TECAN Infinite M200® PRO at 450 nm with a reference wavelength of 650 nm. In order to provide quantitative data, a commercially available mouse anti-human PLA_2_R antibody (abcam, ab211490) was used for standard serial dilutions. Data were obtained with a best-fit standard curve determined by regression analysis using a four-parameter logistic curve fit (4-PL) and expressed as μg/mL.

### 4.18 Statistical analysis

Data were reported as mean ± standard error of the mean (SEM) or number (%). Data analysis was performed using Graph Pad Prism software (Graph Pad, San Diego, CA, USA). All the other data were analyzed using 1-way ANOVA with the Tukey *post hoc* test or the Student t test for unpaired data, as appropriate. Data in **Figures 2B**, **3G**, and **Supplementary Figure S3A** were analyzed by 2-way ANOVA with Tukey *post hoc* test, Šídák’s multiple comparisons test, or Fishers Least Significant Difference test, as appropriate. The statistical significance was defined as a *p-value <*0.05. The sample size for each analysis is indicated in the corresponding figure legend.

## Data Availability

The datasets presented in this study can be found in the online repository Zenodo). (https://zenodo.org/doi/10.5281/zenodo.10660039).
