# Corrigendum: Bi-specific autoantigen-T cell engagers as targeted immunotherapy for autoreactive B cell depletion in autoimmune diseases

**DOI:** 10.3389/fimmu.2024.1543066

**Published:** 2025-01-09

**Authors:** Luca Perico, Federica Casiraghi, Fabiane Sônego, Marta Todeschini, Daniela Corna, Domenico Cerullo, Anna Pezzotta, Patricia Isnard-Petit, Silvia Faravelli, Federico Forneris, Kader Thiam, Ariela Benigni, Giuseppe Remuzzi

**Affiliations:** ^1^ Department of Molecular Medicine, Istituto di Ricerche Farmacologiche Mario Negri IRCCS, Bergamo, Italy; ^2^ Preclinical Models & Services, genOway, Lyon, France; ^3^ The Armenise-Harvard Laboratory of Structural Biology, Department of Biology and Biotechnology, University of Pavia, Pavia, Italy

**Keywords:** autoimmune diseases, membranous nephropathy, anti-PLA2R antibodies, autoreactive B cell, targeted immunotherapies, bi-specific autoantigen-T cell engagers

In the published article, there was an error. Typographical errors have been identified in the **Methods** section pertaining to the description of targeting vector construction for homologous recombination.

A correction has been made to **Materials and methods**, *4.3 Construction of targeting vectors for homologous recombination in embryonic stem cells*. This sentence previously stated:

“The homology arms were isolated by cloning from C57BL/6N mouse genomic DNA. Three targeting vectors were generated targeting specifically Cd3γ, Cd3δ and Cd3ϵ extracellular domains genes in C57BL/6N mouse ES cells. For the Cd3γ targeting vector, the humanizing chimeric CD3γ cDNA was introduced into the murine exon 3 by DNA synthesis. A loxP-flanked puromycin resistance cassette was inserted in 3’ of the humanized cassette. For the Cd3δ targeting vector, the humanizing chimeric CD3δ cDNA was introduced into the murine exon 2 by DNA synthesis. An FRT-flanked hygromycin resistance cassette was inserted in 3’ of the humanized cassette. For the Cd3ϵ targeting vector, the humanizing chimeric CD3ϵ cDNA was introduced into the murine exon 2 by DNA synthesis. A lox2272-flanked neomycin resistance cassette was inserted in 3’ of the humanized cassette. The integrity of the targeting vectors was assessed by full sequencing.”

The corrected sentence appears below:

“The homology arms were isolated by cloning from C57BL/6N mouse genomic DNA. Three different targeting vectors were generated to humanize the three different genes, part of the Cd3 locus: a first vector composed of a CD3γ cDNA upstream of a lox2272-flanked neomycin cassette has been inserted in frame with Cd3γ exon 2; a second vector composed of a CD3δ cDNA upstream of an FRT-flanked hygromycin cassette has been inserted in frame with murine Cd3δ exon 2; a third vector composed of a CD3ϵ complete cDNA upstream of a loxP-flanked puromycin cassette has been inserted in frame with murine Cd3ϵ exon 3. All targeting were performed in cis. The insertion of the human sequences under the control of the mouse promoters and regularity regions prevents the production of the three endogenous mouse Cd3γ, Cd3δ and Cd3ϵ proteins. The integrity of the targeting vectors was assessed by full sequencing.”

The authors apologize for this error and state that this does not change the scientific conclusions of the article in any way. The original article has been updated.

