# Supplementary material for: Bi-specific autoantigen-T cell engagers as targeted immunotherapy for autoreactive B cell depletion in autoimmune diseases
Source: Front Immunol. 2024 Feb 26;15:1335998. doi: 10.3389/fimmu.2024.1335998 (PMC10926275; doi:10.3389/fimmu.2024.1335998)

Supplementary Material

**Supplementary Table S1.** Baseline characteristics of healthy controls in the study group as a whole (Overall) and according to gender.

|  | Overall  (n=6) | Males  (n=3) | Females  (n=3) |
| --- | --- | --- | --- |
| Age (years)* | 36 ± 1 | 37 ± 1 | 35 ± 2 |
| Male sex (%) | 3 (50) | 3 (100) | 0 (0) |

*****mean ± SEM

**Supplementary Table S2.** Baseline characteristics of MN patients in the study group as a whole (Overall) and according to gender.

|  | Overall  (n=6) | Males  (n=3) | Females  (n=3) |
| --- | --- | --- | --- |
| Age (years)* | 54 ± 5 | 55 ± 7 | 53 ± 13 |
| Male sex (%) | 3 (50) | 3 (100) | 0 (0) |
| *Laboratory parameters* |  |  |  |
| Serum creatinine (mg/dL)* | 1.4 ± 0.2 | 1.6 ± 0.4 | 1.2 ± 0.2 |
| Proteinuria (g/24h)* | 10.2 ± 1.6 | 11.0 ± 1.3 | 9.5 ± 3.2 |
| *Disease-related antibodies* |  |  |  |
| Anti-PLA_2_R titer (RU/mL)* | 481.0 ± 138.6 | 486.3 ± 278.1 | 475.7 ± 136.5 |

*****mean ± SEM


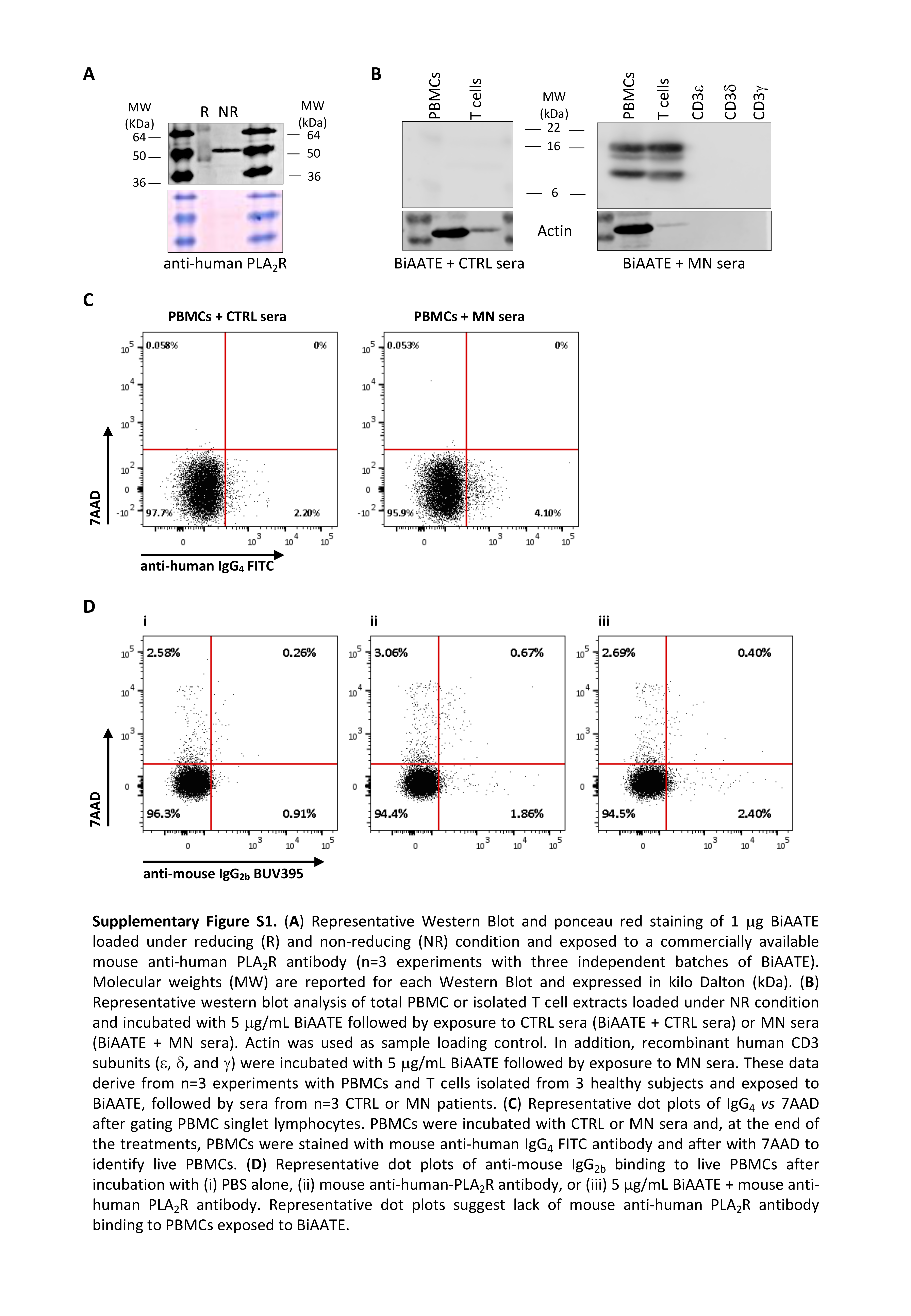


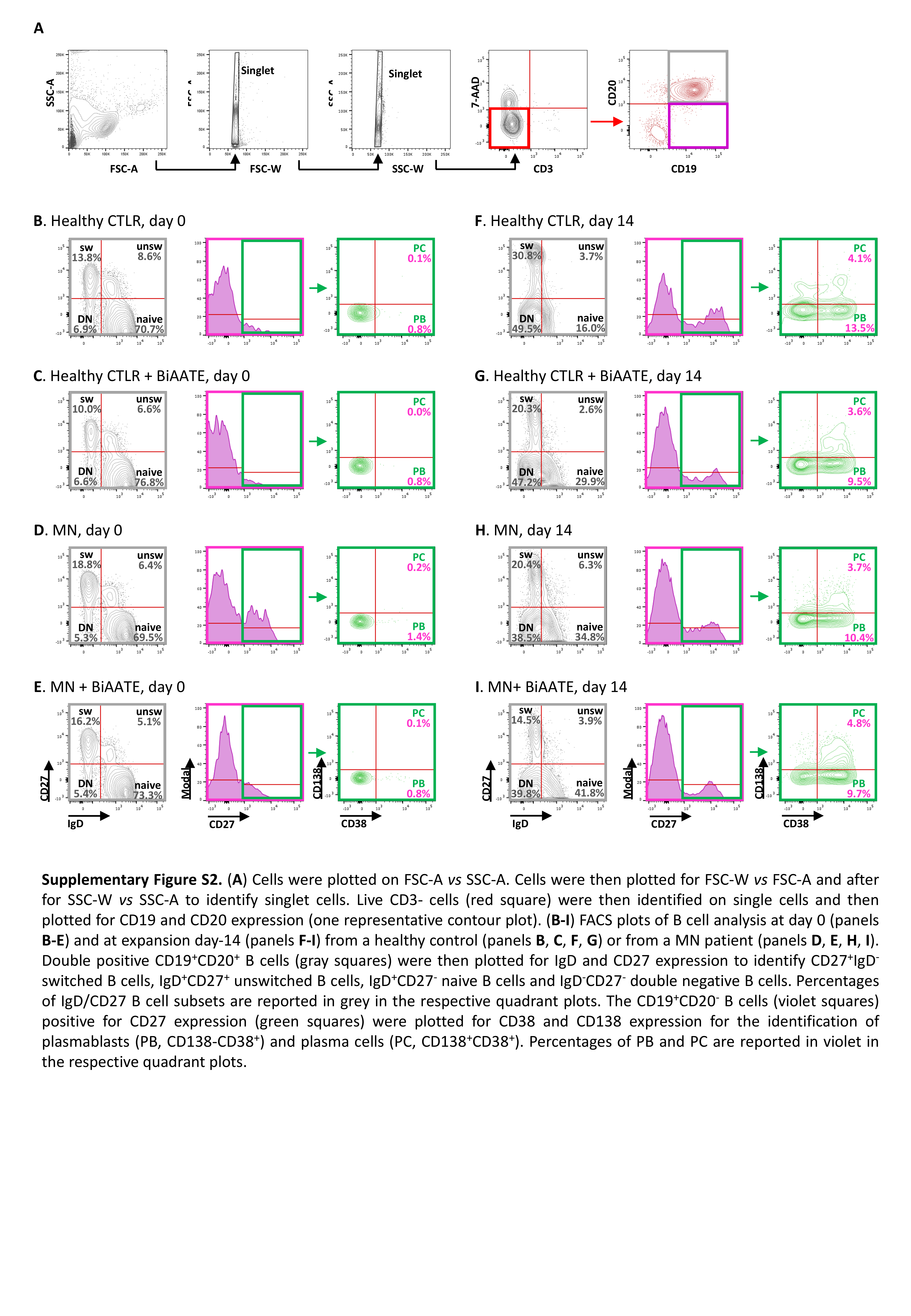


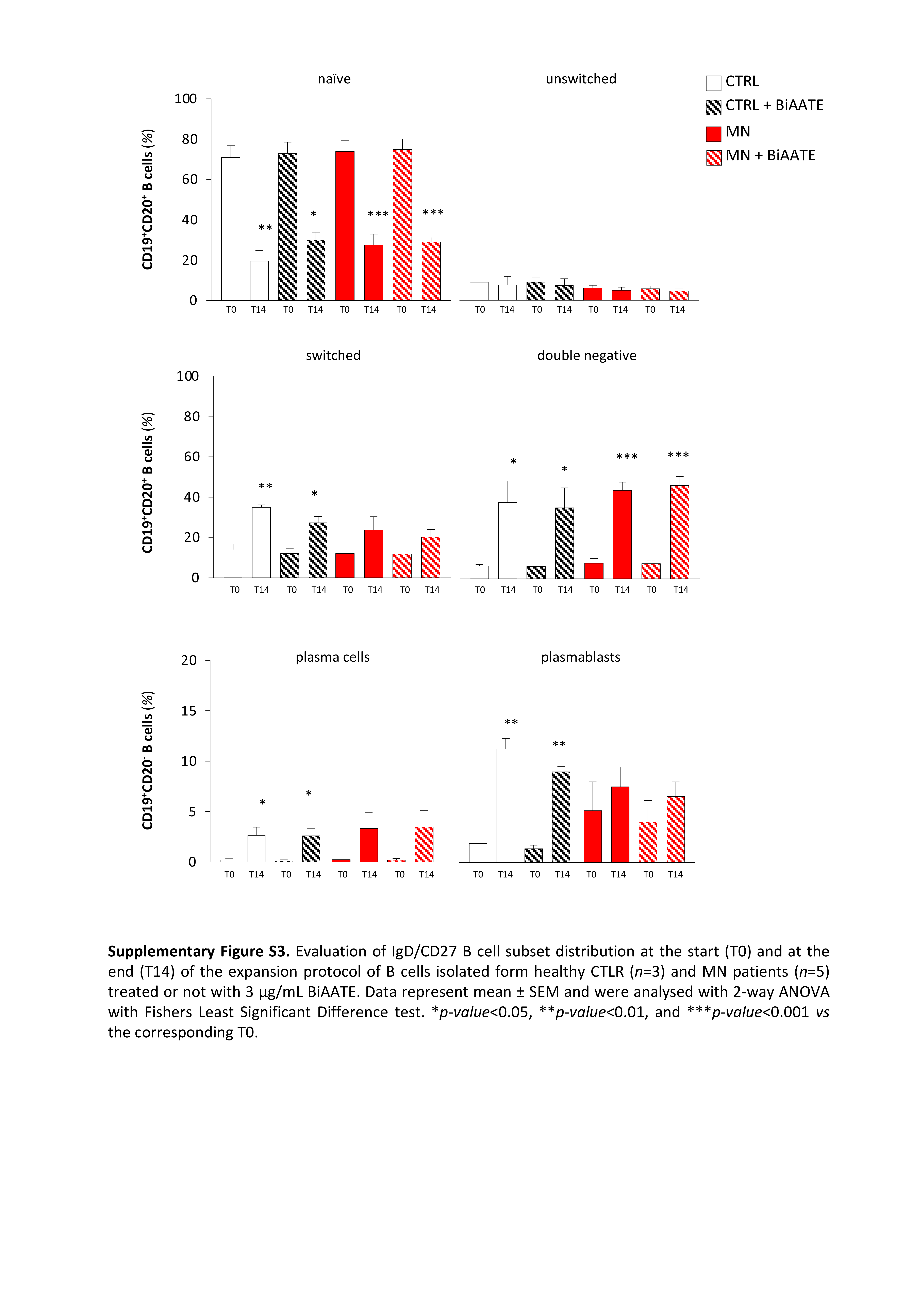


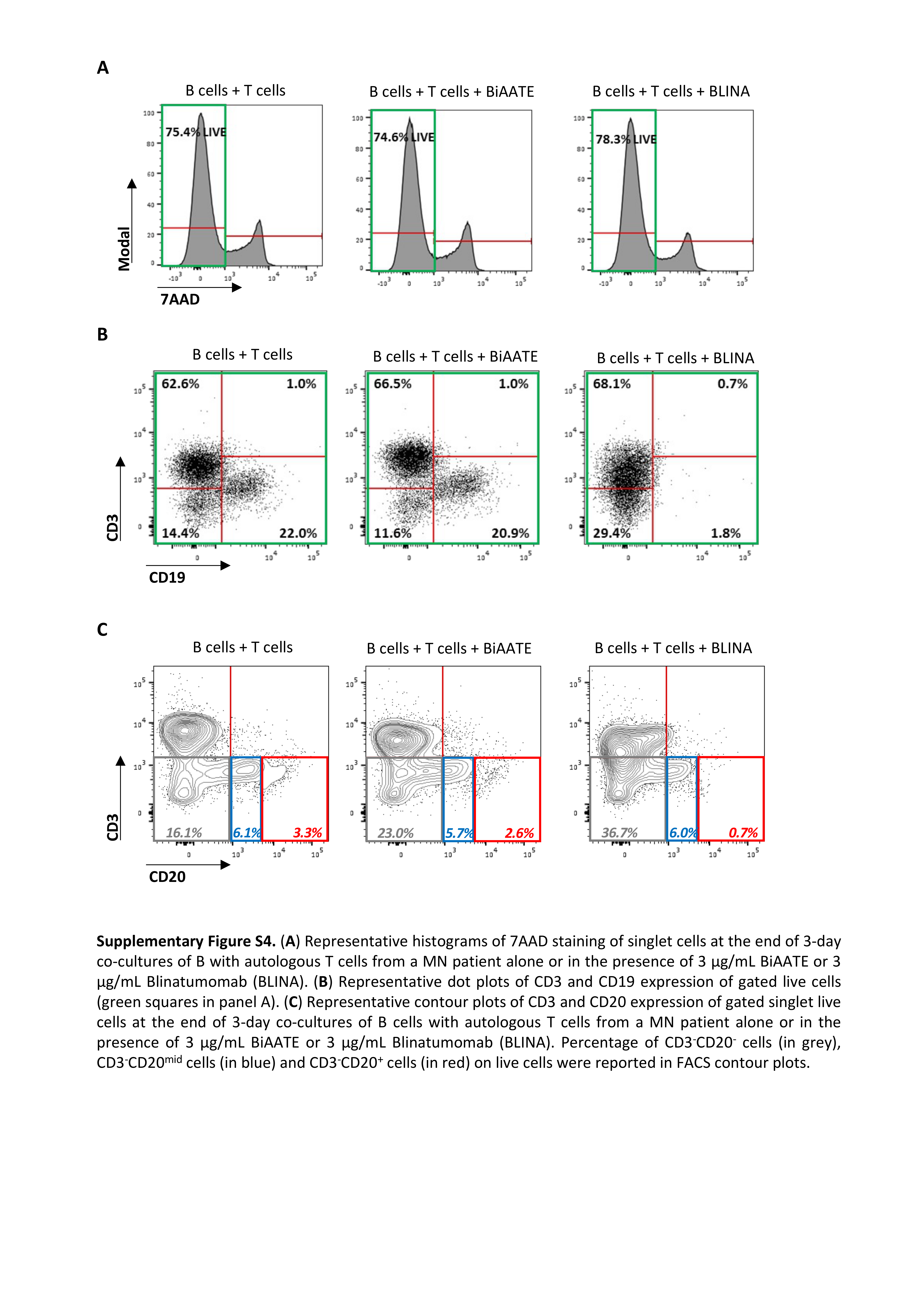


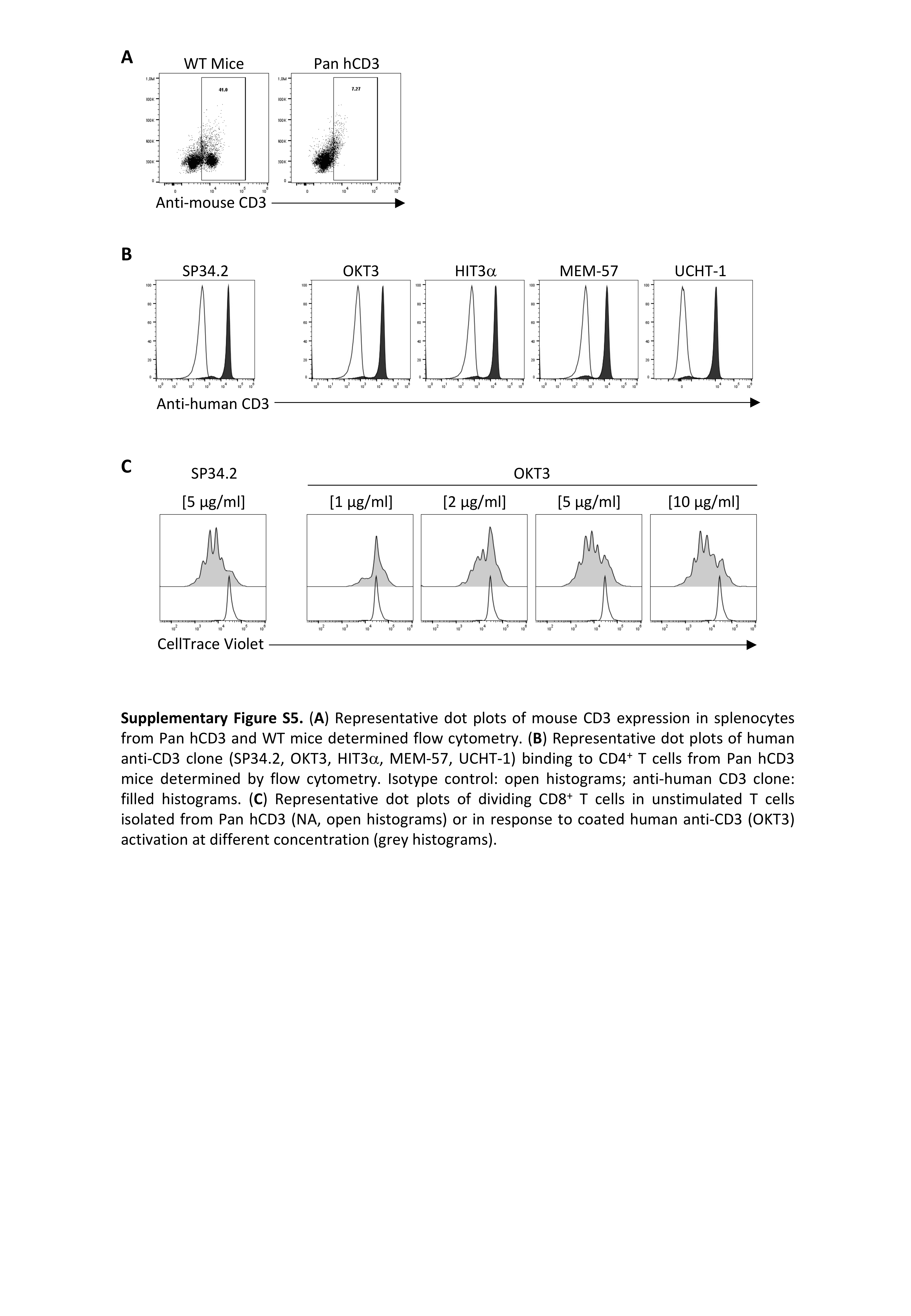


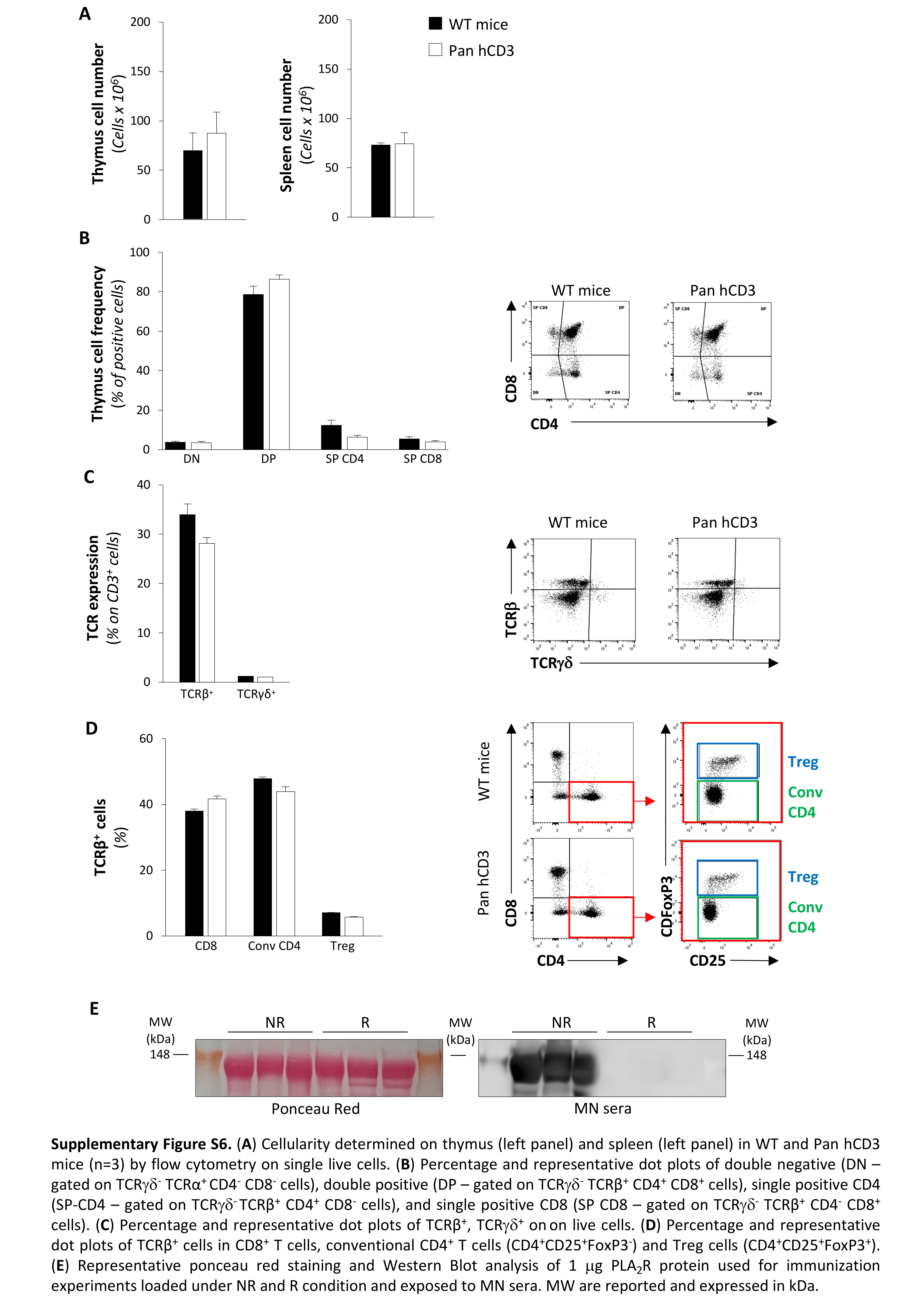

Supplement: Supplementary file 1 [file DataSheet1.docx]
